# An Advanced Compiler Designed for a VLIW DSP for Sensors-Based Systems

**DOI:** 10.3390/s120404466

**Published:** 2012-04-02

**Authors:** Xu Yang, Hu He

**Affiliations:** 1 School of Information and Electronics, Beijing Institute of Technology, Beijing 100081, China; 2 Institute of Microelectronics, Tsinghua University, Beijing 100084, China; E-Mail: hehu@tsinghua.edu.cn

**Keywords:** compilers, optimization, VLIW DSP, sensor-based systems

## Abstract

The VLIW architecture can be exploited to greatly enhance instruction level parallelism, thus it can provide computation power and energy efficiency advantages, which satisfies the requirements of future sensor-based systems. However, as VLIW codes are mainly compiled statically, the performance of a VLIW processor is dominated by the behavior of its compiler. In this paper, we present an advanced compiler designed for a VLIW DSP named Magnolia, which will be used in sensor-based systems. This compiler is based on the Open64 compiler. We have implemented several advanced optimization techniques in the compiler, and fulfilled the O3 level optimization. Benchmarks from the DSPstone test suite are used to verify the compiler. Results show that the code generated by our compiler can make the performance of Magnolia match that of the current state-of-the-art DSP processors.

## 1. Introduction

Nowadays, sensor-based systems are becoming more and more widely used in many domains due to their possibilities in collecting various data from the environment, such as the temperature, humidity, luminosity and many other parameters, and then measuring and otherwise processing it for different purposes. Sensor-based systems are very essential for building useful and fascinating applications that contribute to human life. However, the huge number of emerging applications has imposed strong requirements, such as real-time processing, low-power consumption, reduced size, high-precision algorithms, efficient and secure communications, and many others, on the sensor-based systems, thus increasing demands for architecture improvement and optimization.

Due to their balanced combination of flexibility and hardware performance, Digital Signal Processors (DSPs) have been more and more adopted in sensor-based systems. Large numbers of works have already been announced, in many different application domains.

In early DSPs, instructions were executed in a sequential mode, which means they are executed one after another, having no Instruction-Level-Parallelism (ILP). The drawback is that the resources in the processor cannot be used efficiently, and this probably would lead to poor performance. Several techniques have been proposed to improve the ILP, like superscalar and out-of-order execution.

A superscalar processor dynamically dispatches multiple instructions to parallel functional units, thus enabling execution of more than one instruction during a clock cycle. Out-of-order execution architecture executes instructions in an order different from the one they appear in the program, thus it can make use of clock cycles that would otherwise be wasted by a certain type of costly delay [1].

However, these techniques all come at a cost: increased hardware complexity. Before executing any operations in parallel, the processor must verify that the instructions do not have interdependencies. For example a first instruction's result is used as a second instruction's input. Clearly, they cannot execute at the same time, and the second instruction can't be executed before the first [1].

The Very Long Instruction Word (VLIW) approach, on the other hand, executes instructions in parallel based on a fixed schedule determined when programs are compiled. Since determining the order of execution of instructions (including which instructions can execute simultaneously) is handled by the compiler, the processor does not need the scheduling hardware that the techniques described above require. As a result, VLIW architectures offer significant computational power with less hardware complexity (but greater compiler complexity) than is associated with most superscalar architectures [1].

The VLIW architecture [2] was first reported in 1972 by Joseph Fisher in his research group at Yale University. VLIW architecture typically has multiple functional units (FUs), which means it can execute several instructions in parallel in one clock cycle, thus VLIW can be exploited to greatly improve the ILP.

VLIW architecture is now widely adopted in DSP design, such as in NXP's TriMedia media processors, Analog Devices' SHARC DSP, Texas Instruments' C6000 DSP family, STMicroelectronics' T200 family which based on the Lx architecture, Tensilica's Xtensa LX2 processor, *etc*.

The compiler plays the most important role in the tool-kit of VLIW architecture, as it is in charge of code generation [3]. This paper describes the work of developing an advanced compiler for a VLIW DSP called Magnolia, which is aimed at the sensor-based system application domain. The presented compiler is based on an Open64 compiler, and all four optimization levels of the original Open64 compiler have been achieved. Furthermore, we have implemented several specific optimization techniques in the compiler, to fully exploit the features of the Magnolia processor.

The remainder of the paper is organized as follows: Section 2 provides an overview of the Open64 compiler; Section 3 describes the Magnolia VLIW DSP architecture; Section 4 presents the implementation detail of the Magnolia compiler; benchmark results are given in Section 5; and finally the conclusions are drawn in Section 6.

## 2. Overview of Open64

Open64 [4] is originally derived from the SGI compiler, which is designed for a MIPS R10000 processor, called MIPSPro. It was released under the GNU GPL in 2000, and is an open source, optimizing compiler, which nowadays mainly serves as a research platform for compiler and computer architecture research groups [5,6].

Open64 is written in C++, and supports Fortran 77/95 and C/C++, as well as any combination of these with OpenMP, a shared memory programming API. Open64 is a well-written compiler that performs state-of-the-art analyses, including high-quality inter-procedural analysis, data-flow analysis, data dependence analysis, and array region analysis. Open64 has been proven to generate efficient code for many architectures, including MIPS, ×86, IA-64, ARM, and others [5,6].

Open64 uses an intermediate representation (IR) called Winning Hierarchical Intermediate Representation Language (WHIRL). WHIRL has five different levels (VH, H, M, L, VL), and is used as the common interface among all the front-end and back-end components. Each optimization phase in Open64 is designed to work at a specific level of WHIRL [6]. Open64 is basically composed of five modules: frontends (FE), inter-procedural analysis (IPA), loop nest optimizer (LNO), global optimizer (WOPT), and code generator (CG).

Open64 supports multiple frontends, and can parse C/C++/Fortran programs and translate them into VH level WHIRL. IPA contains two main modules: IPL module (the local part of inter-procedural analysis) and the main IPA module. When IPA is enabled, IPL will be called first. It gathers data flow analysis information from each procedure, and saves the information in files. Then, the main IPA module generates the call graph and performs inter-procedural analysis and transformations based on the call graph. LNO calculates a dependence graph for all array statements inside each loop of the program, and performs loop transformations. WOPT performs aggressive data flow analysis and optimizations based on SSA form. CG creates assembly codes, which will be further transformed to binaries by the assembler [6].

## 3. The Magnolia VLIW DSP Architecture

The target architecture is called Magnolia. It is a VLIW DSP architecture, which is aimed at sensor-based system applications. Magnolia uses the Harvard architecture and load/store address model, and has four different types of functional unit, which are Unit A, Unit M, Unit D and Unit F, respectively. Unit A, Unit M, and Unit D are fixed-point units, while Unit F is floating-point unit. Unit A can execute arithmetic instructions, logical instructions and shift instructions. Unit M can execute multiplication instructions, as well as some arithmetic and logical instructions. Unit D is in charge of memory access and process controlling, as well as some arithmetic and logical instructions. Unit F carries out all the float instructions, including the float vector instructions, too. There are two sets of each functional unit, which means Magnolia has the potential to simultaneously execute eight instructions in one single clock cycle.

**Figure 1.**
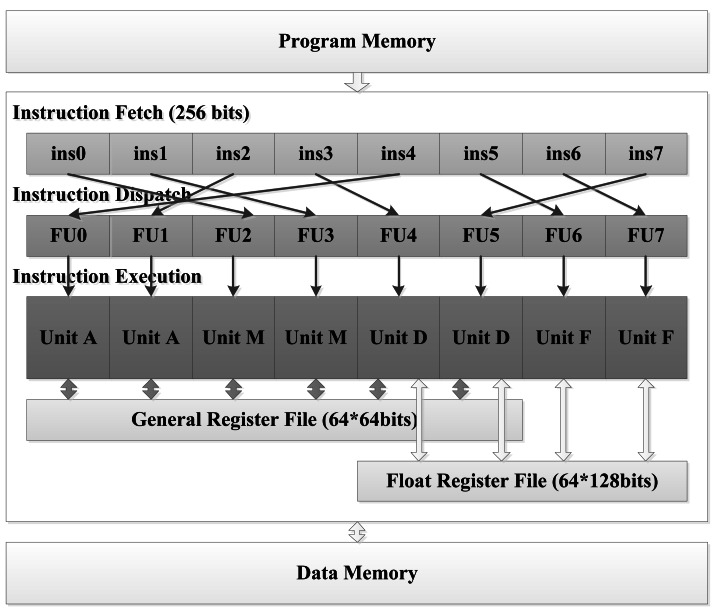
Architecture of Magnolia.

The architecture of Magnolia is shown in Figure 1. The processor can be roughly divided into three parts: the instruction fetch unit, the instruction dispatch unit and the instruction execution unit. The width of instruction of the Magnolia architecture is 32 bits. The instruction fetch unit gets eight instructions from the program memory at one time. The instruction dispatch unit judges and determines the execution packet, and dispatches the instructions to the corresponding functional unit.

The pipeline of Magnolia has 10 stages, where four stages belong to the instructions fetch unit, one stage belongs to the instruction dispatch unit, and two to five stages belong to the instruction execution unit, according to the instruction type.

The general register file has 64 registers, each of which has 64 bits, and can be accessed by Unit A, Unit M and Unit D. The float register file also has 64 registers. Every float register is 128 bits, and can be accessed only by Unit D and Unit F. So, Unit D is responsible for the data conversion between integer data and float data.

Magnolia's instruction set has independent intellectual property rights. Vector instructions and specific instructions are included in the instruction set, to facilitate DSP applications. The whole instruction set can be mainly divided into two categories, the fixed-point instructions and the float-point instructions. The fixed-point instruction category contains arithmetic instructions, logic instructions, shift/rotate instructions, multiplication instructions, data movement instructions, compare instructions, sorting instructions, control instructions and vector instructions. The floating point instruction category contains arithmetic instructions, logic instructions, compare instructions, data conversion instructions, data movement instructions, sorting instructions, and vector instructions.

## 4. The Implementation of the Magnolia Compiler

The responsibility of a VLIW compiler includes: (1) determining the correctness of the syntax of programs, (2) generating correct and efficient object code, (3) run-time organization, and (4) formatting output according to assembler and/or linker conventions [7].

**Figure 2.**
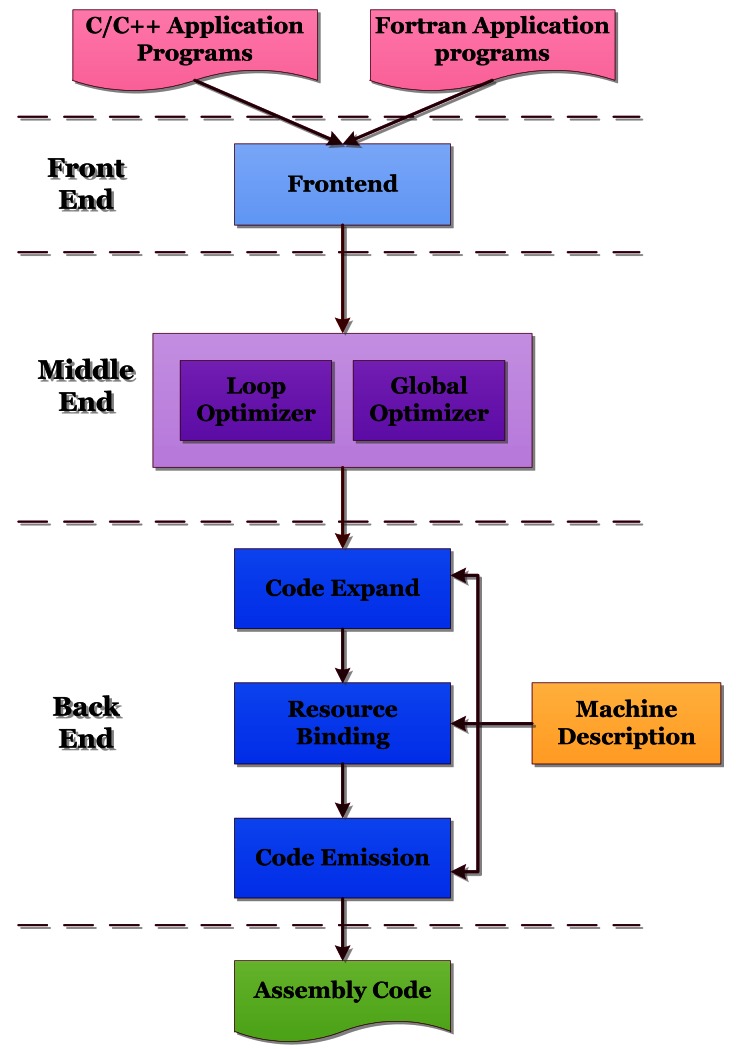
Architecture of the Magnolia compiler.

The architecture of our Magnolia compiler is illustrated in Figure 2. It can be divided into three main parts: the frontend, the middle end, and the backend. The front end checks the programming language syntax and semantics, and generates an Intermediate Representation (IR) of the source code. The middle end performs optimizations which are mostly independent of the underling hardware. The back end translates the IR further into assembly code. There are four optimization levels, which are O0, O1, O2 and O3, respectively, in the Magnolia compiler. We will go through each in more details in the following sections.

### 4.1. Front End

The frontend stage of Magnolia compiler takes application programs written in C/C++/Fortran languages as input, performs syntax and semantics, and translates the programs into High Level WHIRL.

### 4.2. Middle End

The middle end of Magnolia compiler is mainly composed of two parts: loop optimizer and global optimizer, which perform code optimization during the lowering and transformation of WHIRL code. When the optimization level is below O2, the various optimization techniques used in the middle end will not work. It will only perform WHIRL code lowering and transformation.

#### 4.2.1. Loop Optimizer

The loop optimizer performs transformation on loops to optimize the compile code. Loop optimizer is called only when the optimization level is O3. It works on High Level WHIRL, and removes unstructured control flow elements, such as goto and switch. The loop optimizer is driven by the analysis of data dependence. It analyzes extract information from WHIRL, and constructs specific Intermediate Representations.

The pre-optimization module is called first, to analyze the data dependence, and prepare for the main process. Then, in the main process, the loop optimizer performs several loop target optimization techniques, such as loop unswitching, cache blocking, loop fission and fusion, parallelization, loop interchange, soft prefetch, loop split, and software pipeline. We also implemented an auto-vectorization module in the loop optimizer to enhance the data manipulation ability of our compiler.

A lot of existing approaches in research perform auto-vectorization at a late stage of the compilation process, *i.e.*, in the backend, because more information is available at the backend, such as a more precise data flow of the input program and the info about the underling target hardware. However, the disadvantage is that the data parallelism in loops cannot be effectively exploited by these techniques, so the code quality can be less optimal.

In this work, we implemented a high level auto-vectorization module to generate SIMD code by examining the loop code. The auto-vectorization module is in the early stage of the compilation process, just after the input source code program has been transformed into the intermediate presentation (IR). As this approach only needs simple knowledge of the target machine's instruction set architecture, it is easily retargetable. The data packing work is done in the same time as the SIMD code is generated, thus, it can ease the work of register allocation in the backend stage. Our auto-vectorization module is also compatible with all the state-of-the-art code optimization techniques in the Magnolia compiler.

As in the real application programs, the loop form can be various, the iteration index might be implicit, the step that the iteration index changes might be irregular, and so on. These features sometimes might be difficult for the compiler to analyze for generating the SIMD code. In order to ensure the quality of the auto-vectorization module, we have developed several rules to limit the loop form that suit our approach, so the preprocessing process is involved to make sure that only the loop satisfies the rules is picked to perform further auto-vectorization.

We define these rules mainly for finding the manifest loop to perform auto-vectorization, and nine rules are defined in total. The loops that satisfy the principles will be analyzed, to identify operations that can be vectorized.

The main stage of the auto-vectorization module in the loop optimizer can be roughly divided into two parts. First, the approach prepares the data for the operations that can be vectorized, including the process of data alignment and memory access. Then, the loop is unrolled, and the identified operations are transformed into vectorized presentations in the IR, while the IR is also reconstructed.

In the code expansion module of back-end, the compiler will carefully map the vectorized operations in the IR to the right vector instructions in the Magnolia instruction set.

#### 4.2.2. Global Optimizer

The global optimizer works on the Medium Level WHIRL. Depending on the level of optimization, the global optimizer may be invoked multiple times in the same program unit during different compiler phases.

When the optimization level is below O2, the global optimizer is not involved. On optimization level O2, the global optimizer is invoked just before the back end of the Magnolia compiler. It performs its full set of optimizations and generates alias info for the back end. At optimization level O3, the global optimizer is also invoked by the loop optimizer. It will generate def-use and alias info for loop optimizer.

The global optimizer uses Static Single Assignment (SSA) as the program representation. It creates the dominator tree, post-dominator tree, dominance frontier, as well as computing SSA form and the control-dependence set of the Control Flow Graph (CFG). It then performs def-use analysis, alias classification and pointer analysis, induction variable recognition and elimination, copy propagation, dead code elimination, partial redundancy elimination and more. Finally, it transforms the SSA form back to Very Low Level WHIRL after these analyses and optimizations.

### 4.3. Back End

The back end of Magnolia compiler is composed of three phases: Code Expand, Resource Binding, and Code Emission.

The Code Expand phase transforms the Very Low level WHIRL from middle end into Code Generator Machine Instruction Representation (CGIR) [5,6], which is the intermediate representation form used in the back end of Magnolia compiler. It grouped the operations in the Very Low level WHIRL intermediate representation into regions and basic blocks, and translated them into instructions.

In the Resource Binding phase, these instructions are bidden to certain machine resources, such as clock cycle and functional unit. Also, variables used by the instructions are allocated to registers or memories, too. And data access instructions are generated accordingly.

Finally, in the Code Emission phase, the compiler transforms CGIR into assembly format, and emits the code.

These three phases in the back end of Magnolia compiler are all hardware architecture dependent. In Open64, the info about the underlying machine architecture is described in the Machine Description Files, which is separate from the compile code. The hardware info that is used in the back end phases will be generated during the establish course of the complier. It provides a convenient way for the compiler to understand the underlying machine architecture features, and also a means to enhance the portability of the compiler code. We adopt this pattern in the Magnolia compiler.

#### 4.3.1. Machine Description

The characteristics of the target machine architecture that the compiler needs to know can be divided into three major groups: the instruction set architecture (ISA), the application binary interface (ABI), and the processor model.

ISA is the part of the computer architecture related to programming, and the specification of the set of opcodes (instructions). We need to describe the whole instruction set, including the names of the instructions, the functions of the instructions, the numbers of the operand for each instruction, the data types and store types of the operand for each instruction, the assembly code format of each instruction, and other properties of each instruction. Also, we need to define the native data types, registers, addressing modes, and memory architecture.

ABI describes the interface between an application program and the libraries or other parts of the application program, and covers details such as data type, data size, data alignment, and the calling convention, which controls how functions' arguments are passed and return values retrieved.

The processor model defines the data path of the target machine, such as the type of functional units, the number of different types of functional units, and the number of instructions can be issued in one clock cycle. The processor model also includes the execution details of instructions, like the execution latency of each instruction, the time required to prepare the data for each instruction, and the resources needed to carry out a certain instruction.

#### 4.3.2. Code Expansion

The task of the Code Expansion phase is to replace the operations in IR with the actual instructions from the instruction set of the target machine. The algorithm used in this phase is similar to the one used in the Open64 compiler. However, as there are some specific instructions in the Magnolia instruction set, designed to enhance the ability of signal processing, such as shuffle instruction, butterfly instruction, and branch instruction with delay slots, which would be very useful in the sensor-based systems, several optimization techniques are involved, to facilitate the usage and mapping of these specific signal processing target instructions.

#### 4.3.3. Resource Binding

**Figure 3.**
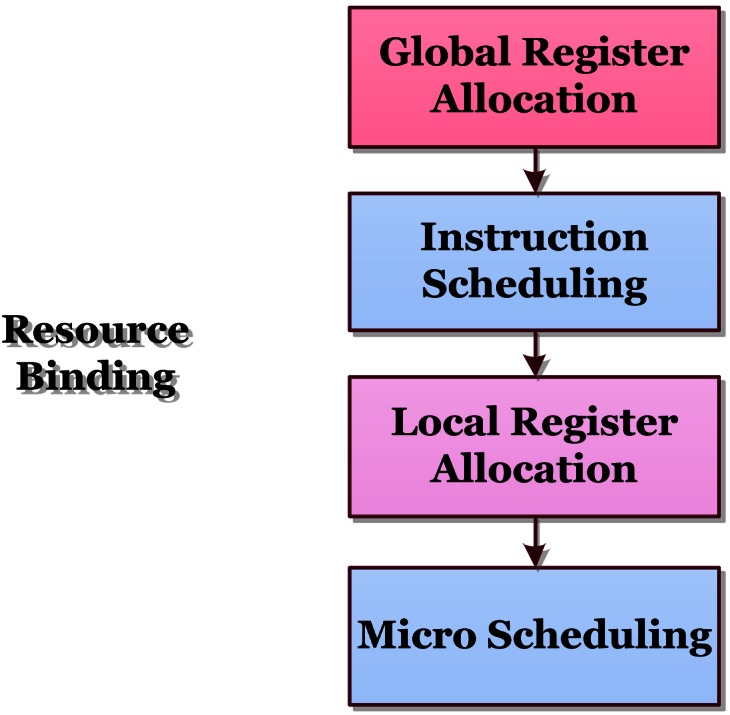
Illustration of the Resource Binding phase.

The Resource Binding phase is responsible for two major tasks: instruction scheduling and register allocation. There are four major modules in this phase, which is illustrated in Figure 3.

First, the global register allocation module is called, which performs some early global variable related optimization over a whole function/procedure level, to improve the localization of data, thus enhancing the effectiveness of local register allocation. Then, the instruction scheduling module performs the main scheduling for instruction. After that, the local register allocation module calculates living period for variables, and allocates registers for them. Finally, as the ubiquitous problem existed in VLIW architectures that the pressure of register allocation might cause some conflicts in the scheduling decisions of instruction scheduling, the micro scheduling module is called to perform some necessary fine-grained instruction scheduling decision adjustment and optimization, to solve those conflicts.

It must be mentioned that the global register allocation module is invoked only when the optimization level is above O2. The instruction scheduling module and the micro scheduling module are enabled only when the optimization level is above O1.

As there are multiple functional units in the VLIW architecture, a major task for a VLIW compiler is to exploit the instruction level parallelism as thoroughly as possible. This means that a VLIW compiler must enhance its ability for data manipulation and program manipulation. Data manipulation refers to the ability to arrange data into certain patterns, so that the compiler can exploit vector instructions to enhance the ILP. Program manipulation lets the compiler to pack instructions as many as possible into one cycle, so that the ILP is enhanced.

In this paper, the data manipulation ability of our compiler is improved by implementing an auto-vectorization module in the loop optimizer, which is discussed in Section 4.2.1. The program manipulation ability is enhanced through improvement on the original Open64 scheduling algorithm.

In the Magnolia processor, most instructions can be executed by different types of functional units, providing more chances to develop the ILP, which finally leads to performance enhancement, and can greatly improve the fine-grained flexibility of instruction scheduling decisions.

The main procedure of the clock cycle and functional unit binding of instructions is finished in the instruction scheduling module. However, the register allocation stage might bring in some additional load/store instructions to deal with the memory accesses due to the lack of enough registers, so the micro scheduling stage is involved to bind these additional load/store instructions to properly resources, and to make fine-grained adjustment of the instruction scheduling decision by the instructions scheduling stage to further improve the code.

In the VLIW architecture, there are multiple functional units, and up to eight instructions could be executed in one clock cycle, so the pressure on registers might be an issue. In the global register allocation stage, some preliminary optimization of the variables, such as breaking up the global variables into localized ones, is performed to facilitate the task of the local register allocation stage.

In the local register allocation stage, first, the living period of each variable is calculated. Then, all the living periods are analyzed, and they are split or combined to match the available register resources. The basic graph coloring model is sufficient, as the register hierarchy of Magnolia is very simple. If registers are not enough, additional store and load instructions must be created and inserted into the original instruction queue, to spill the data of a symbolic register to memory and restore it to a register later when need to be used. Accessing memory is much slower than accessing registers, and this would slow down the execution speed. As in VLIW architectures, the compiler needs to maximize the ILP, thus it needs to put as many instructions as possible be executed in parallel, which might impose large demands on register numbers, meaning spilling often happens.

A specific spill register file is included in Magnolia architecture. In common cases, when there are not enough registers, the compiler must spill some data into memories, to make some spare registers. These data would need to be restored from memories into register later when they are needed. The access to memories is very slow compared to the computing of the processor, so the performance of the processor would be harmed. Thus, we introduced the spill register file mechanism. In the Magnolia compiler, when spilling happens, the data is first transferred into a spill register file, and restored from the spill register file into the general register when needed. Only when the spill register file is full too, does the compiler need to spill data into memories. This mechanism can substantially enhance the performance, as proven by our experiment results shown in Section 5.2.

There are some other optimization-modules in the Resource Binding phase specific designed for the Magnolia architecture, for example, the code optimization module for branch instruction. In the code expansion phase, the Magnolia compiler generates classic branch instructions, which would cause the cleaning up of instructions in the pipeline stages after the branch instruction when it is executed. Then, after the Resource Binding phase is over, an optimization module would be called to check through the instruction queue to identify the opportunities where classic branch instruction can be transformed into branch instruction with delay slot, and perform the transformation. As branch instructions with delay slots do not need to clean up the pipeline stages, this would help to enhance the performance. Also, the Magnolia compiler supports several addressing modes for load and store operations, which is quite useful in the DSP domain.

#### 4.3.4. Code Emission

The Code Emission phase transforms the CGIR intermediate representation into assembly format, and emits the assembly code. The assembly format of Magnolia is described in the Machine Description Files.

It must be mentioned that, according to the definition of the Magnolia assembly format, the instructions which are executed paralleled in one clock cycle, must be arranged in a certain pattern where the functional units of these instructions are in an ascending order. Otherwise, the assembler cannot identify the instruction parallelism correctly. Thus, in Code Emission phase, our Magnolia compiler must perform an additional task, that is to check and rearrange the issue order of instructions, so that the information about the instruction parallelism can be delivered to the assembler in a right way.

## 5. Results and Discussion

### 5.1. Experimental Framework

The class of applications used for experiments belongs to the DSP-stone [8] benchmark suite. Those benchmarks in the DSP-stone benchmark suite, such as matrix, fir, lms, and fft, represent quite a broad spectrum of the possibilities of using DSP in a sensor-based system. As development of the Magnolia processor is still in progress, we only evaluated the performance of running the compiled code on a simulator designed for Magnolia, which is based on the gem5 [9] simulator.

### 5.2. Results and Discussion

**Figure 4.**
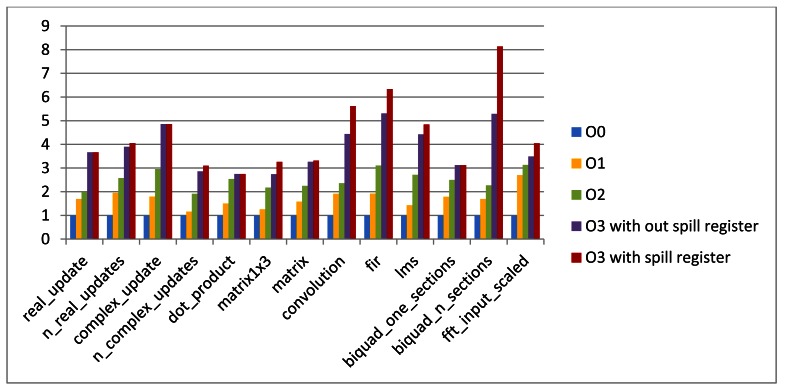
Performance results.

Experimental results of the DSP-stone benchmark suite running on the simulator are normalized and presented in Figure 4.

Blue bar showed the performance (measured by the number of execution cycles) generated by the compiler on optimization level O0, without any optimization and instruction scheduling. Orange bars showed the performance generated by the compiler on optimization level O1, which involves instruction scheduling, but still not any optimization. Green bars show the performance generated by the compiler on optimization level O2, involving the global optimizer. Purple bars show the performance generated by the compiler on optimization level O3, with all the optimizations, but without using the spill register file. Red bars show the performance generated by the compiler on optimization level O3, with all the optimizations and using the spill register file. These results indicated that with all the optimization techniques working, the performance gain can be around 4.39 times on average against to the code generated without any optimization.

It can be concluded from the result comparison between optimization level O0 and optimization O1 that, as there are large amount of functional units in the VLIW architecture, if the ILP of applications can be well developed by the compiler through instruction scheduling, then the performance enhancement can be very large. However, if the application does not have much ILP in itself, then the enhancement is small. As for optimization level O2, the global optimizer phase and the global register allocation stage in resource binding phase are involved. Those optimization techniques in these stages further improved the performance. The optimization techniques invoked in optimization level O3 are mainly focused on loop optimization, thus the optimization effectiveness can be very significant for applications with large fractions of loop structures.

The spill register file in this experiment is set to be composed of 64 registers, each of which has 64 bits, and can be accessed only by Unit D. It can be drawn from the picture that, when the spill register file is used, as the access time to the memories is largely reduced, the performance can be further improvement. However, in case of the register pressure is not so serious, so the performance enhancement by the spill register file might be less significant. It must be mentioned that, the implementation of the spill register file in the Magnolia architecture would need additional area. However, compared to the performance enhancement brought by the spill register mechanism, this penalty can be acceptable. Also, the number of registers in the spill register file can be well designed to achieve a trade-off between the chip size increase and the performance enhancement according to the specific sensor application domains. The compiling overhead for Magnolia compiler is very small, and there is very little variation between different optimization levels.

## 6. Conclusions

In this paper, we have presented an advanced compiler designed for a sensor-based system application oriented VLIW DSP called Magnolia. The compiler is designed based on the Open64 compiler. We have implemented the original optimization techniques of the Open64 compiler in the Magnolia compiler, and also added some specific techniques which can help exploit the Magnolia processor's potential for digital single processing, such as the auto-vectorization module, and the support for spill register files. The compiler is evaluated using the DSP-stone benchmark suite. The results show our optimization techniques can bring about a large performance improvement.
